# Co-existence of virulence factors and antibiotic resistance in new *Klebsiella pneumoniae* clones emerging in south of Italy

**DOI:** 10.1186/s12879-019-4565-3

**Published:** 2019-11-04

**Authors:** Teresa Fasciana, Bernardina Gentile, Maria Aquilina, Andrea Ciammaruconi, Chiara Mascarella, Anna Anselmo, Antonella Fortunato, Silvia Fillo, Giancarlo Petralito, Florigio Lista, Anna Giammanco

**Affiliations:** 1 Department of Health Promotion, Mother and Child Care, Internal Medicine and Medical Specialties, University of Palermo, Via del Vespro 133, 90127 Palermo, Italy; 2 Scientific Department, Army Medical Center, Via S. Stefano Rotondo, 4 - 00184 Rome, Italy

**Keywords:** Carbapenem-resistant *Klebsiella pneumoniae*, Virulence factors

## Abstract

**Background:**

Endemic presence of *Klebsiella pneumoniae* resistant to carbapenem in Italy has been due principally to the clonal expansion of CC258 isolates; however, recent studies suggest an ongoing epidemiological change in this geographical area.

**Methods:**

50 *K. pneumoniae* strains, 25 carbapenem-resistant (CR-Kp) and 25 susceptible (CS-Kp), collected from march 2014 to march 2016 at the Laboratory of Bacteriology of the Paolo Giaccone Polyclinic University hospital of Palermo, Italy, were characterized for antibiotic susceptibility and fully sequenced by next generation sequencing (NGS) for the in silico analysis of resistome, virulome, multi-locus sequence typing (MLST) and core single nucleotide polymorphism (SNP) genotypes

**Results:**

MLST in silico analysis of CR-Kp showed that 52% of isolates belonged to CC258, followed by ST395 (12%), ST307 (12%), ST392 (8%), ST348 (8%), ST405 (4%) and ST101 (4%). In the CS-Kp group, the most represented isolate was ST405 (20%), followed by ST392 and ST15 (12%), ST395, ST307 and ST1727 (8%). The in silico β-lactamase analysis of the CR-Kp group showed that the most detected gene was *bla*SHV (100%), followed by *bla*TEM (92%), *bla*KPC (88%), *bla*OXA (88%) and *bla*CTX-M (32%). The virulome analysis detected *mrk* operon in all studied isolates, and *wzi-2* was found in three CR-Kp isolates (12%). Furthermore, the distribution of virulence genes encoding for the yersiniabactin system, its receptor *fyu*A and the aerobactin system did not show significant distribution differences between CR-Kp and CS-Kp, whereas the *Klebsiella* ferrous iron uptake system (*kfu*A, *kfu*B and *kfu*C genes), the two-component system *kvg*AS and the microcin E495 were significantly (*p* < 0.05) prevalent in the CS-Kp group compared to the CR-Kp group.

Core SNP genotyping, correlating with the MLST data, allowed greater strain tracking and discrimination than MLST analysis.

**Conclusions:**

Our data support the idea that an epidemiological change is ongoing in the Palermo area (Sicily, Italy). In addition, our analysis revealed the co-existence of antibiotic resistance and virulence factors in CR-Kp isolates; this characteristic should be considered for future genomic surveillance studies.

## Background

The World Health Organization (WHO), the US Centers for Disease Control and Prevention (CDC) and the UK Department of Health have indicated *Klebsiella pneumoniae* as one of the multi drug resistant (MDR) microorganisms constituting an immediate threat for human health [1–3]*. K. pneumoniae*, an opportunistic pathogen, has emerged not only thanks to its ability to accumulate multiclass antibiotic resistance determinants over time [1] but also, as widely reported, to its adeptness in causing severe community- and hospital-associated infections [4–6].

*K. pneumoniae* “permeability” to mobile genetic elements is a key factor for its dissemination not only with respect to the possibility of becoming resistant to antibiotics, but also of evolving towards more virulent phenotypes thanks to genes that may provide a survival benefit to microorganisms [7, 8]. However, in *K. pneumoniae* the relation between resistance and virulence is a complex issue since a systematic understanding of its population structure is still lacking [4, 9]. This makes it difficult to perceive the emergence of new clones, what instead could be an advantageous approach to develop epidemiological surveillance programs and avoid outbreaks, particularly of strains which have become resistant to carbapenem (carbapenem-resistant *K. pneumoniae*, CR-Kp) [9–11].

The existing body of research on carbapenem-resistance mechanisms suggests that the production of *K. pneumoniae* carbapenemase (KPC) encoded by the plasmidic gene *bla*KPC is the most common one and its rapid dissemination has typically been caused by the clonal expansion of clonal complex (CC) 258 strains, including ST258 and ST512 [12–16]. In Italy, the first KPC-positive *K. pneumoniae,* belonging to ST258, was isolated in Florence in 2008 [17]. Since then, the diffusion of these strains has been evident. In fact, the last European Antimicrobial Resistance Surveillance Network report has confirmed an average prevalence of CR-Kp of up to 33.9%, percentages that make Italy an endemic country for this microorganism [18, 19].

To date, although some research has been carried out on the diffusion and genetic characteristics of KPC-positive *K. pneumoniae* in our region (Sicily, Italy), no single study exists which has comprehensively described these strains considering all of the Hospital’s Departments for a period longer than 1 year [20–24].

In this study, we fully sequenced 50 *K. pneumoniae* strains, both carbapenem resistant and -susceptible, collected from March 2014 to March 2016 at the Laboratory of Bacteriology of the Department of Sciences for Health Promotion and Mother-Child Care “G. D’Alessandro” (Paolo Giaccone Polyclinic University Hospital, University of Palermo, Italy).

The primary aim of this study was to take a current snapshot of the distribution of *K. pneumoniae* in our geographic area by: 1) characterizing the virulome and resistome of CR-Kp clones; 2) assessing the extent to which virulence determinants were carried by CR-Kp and CS-Kp (carbapenem susceptible *K. pneumoniae*); 3) investigating the phylogenetic correlations among samples by Multilocus sequence typing (MLST) in silico and the analysis of core single nucleotide polymorphisms (SNPs).

## Methods

### Bacterial strains and antimicrobial susceptibility testing

Species and antimicrobial susceptibility were determined using the Becton- Dickinson Phoenix™ automated system (Becton Dickinson, Sparks, MD, USA). Resistance to carbapenem was established by interpreting the results of the antimicrobial susceptibility test on the basis of the breakpoint criteria of the European Committee on Antimicrobial Susceptibility Testing [25]. On the base of carbapenem susceptibility the 50 strains were divided in 25 isolates resistant (CR-Kp) and 25 sensitive (CS-Kp). Table 1 shows the entire clinical sample and the departments of isolation.

**Table 1:** *K. pneumoniae* CR and CS isolates, department of isolations, host disease, host age and clinical sample

CR-Kp ID	Department	host disease	host age	Sample	CS-Kp ID	Department	host disease	host age	Sample
1 R	General surgeries emergencies	sepsi	70	Blood PVC	1 S	Endocrinology and Metabolic diseases	ICU	75	Urine
2 R	Anaesthesia and resuscitation	sepsi	46	Blood CVC	2 S	Surgical Oncology	ICU	81	Urine
3 R	Anaesthesia and resuscitation	sepsi	59	Blood PVC	3 S	Nephrology and Hypertension	ICU	70	Urine
4 R	Anaesthesia and resuscitation	pneumoniae	60	Bronchoalveolar lavage	4 S	Clinical Respiratory Medicine	ICU	64	Urine
5 R	General surgeries emergencies	sepsi	36	Intra-abdominal fluid	5 S	General and Thoracic Surgery	ICU	52	Wound swab
6 R	Internal Medicine Cardioangiology	sepsi	36	Blood PVC	6 S	Geriatric medicine	infected wound	21	Urine
7 R	Cardiac surgery	sepsi	53	Blood CVC	7 S	Geriatric medicine	ICU	82	Ulcer swab
8 R	Clinical Respiratory Medicine	ICU	77	Urine	8 S	Plastic surgery	infected wound	82	Tissue
9 R	Internal Medicine	ICU	49	Urine	9 S	Clinical Respiratory Medicine	infected wound	74	Urine
10 R	Clinical Respiratory Medicine	ICU	79	Urine	10 S	Internal Medicine Cardioangiology	ICU	76	Sputum
11 R	Geriatric medicine	ICU	90	Urine	11 S	Infectious disease	pneumoniae	77	Sputum
12 R	Endocrinology and Metabolic diseases	ICU	84	Urine	12 S	Clinical Respiratory Medicine	pneumoniae	22	Bronchoalveolar lavage
13 R	Anaesthesia and resuscitation	sepsi	70	Blood PVC	13 S	NICU	pneumoniae	81	Endotracheal tube
14 R	Internal Medicine Cardioangiology	sepsi	54	CVC	14 S	Haematology and Bone Marrow Transplantation	pneumoniae	15 days	Sputum
15 R	Cardiac surgery	pneumoniae	72	Bronchoalveolar lavage	15 S	Rheumatology	pneumoniae	83	Urine
16 R	Anaesthesia and resuscitation	ICU	60	Urine	16 S	Internal Medicine	ICU	73	Sputum
17 R	Internal Medicine Cardioangiology	infected wound	38	Ulcer swab	17 S	Geriatric medicine	pneumoniae	76	Urine
18 R	Internal Medicine Cardioangiology	pneumoniae	77	Sputum	18 S	Rheumatology	ICU	36	Urine
19R	Cardiac surgery	infected wound	71	Wound swab	19 S	Clinical Respiratory Medicine	ICU	91	Sputum
20 R	Clinical Respiratory Medicine	ICU	80	Urine	20 S	General surgeries emergencies	pneumoniae	63	Liquor
21 R	General surgeries emergencies	bile infections	65	Bile	21 S	Internal Medicine Cardioangiology	Meningitidis	92	Urine
22 R	Anaesthesia and resuscitation	sepsi	70	Blood CVC	22 S	Anaesthesia and resuscitation	ICU	82	Urine
23 R	Cardiac surgery	pneumoniae	62	Sputum	23 S	Haematology and Bone Marrow Transplantation	ICU	70	Cutaneous swab
24R	General surgeries emergencies	sepsi	72	Intra-abdominal fluid	24 S	Tourism and Migration	infected wound	81	Urine
25 R	General surgeries emergencies	sepsi	54	Abscess fluid	25 S	Internal Medicine Cardioangiology	ICU	71	Urine

CVC: central venous catheter, ICU: intensive care unit, NICU: neonatal intensive care unit, PVC: Peripheral venous catheter

### DNA isolation

The template DNA was prepared from bacterial colonies grown for 18 h on Blood Agar plates. Colonies were picked and suspended in 500 μl of ultra-pure DNase-free water. The suspension was harvested at 14000 rpm for 10 min. The supernatants were discarded while DNA from the pellets were extracted using the QIAmp® DNA Mini kit *Qiagen* (QIAGEN; Hilden, Germany), the quantity and purity of the DNA were determined using NanoDrop 8000 spectrophotometer (Thermo Fisher Scientific, Waltham).

### Whole-genome sequencing

Isolate’s genomes were fully sequenced at the Scientific Department, Army Medical Center, Military Polyclinic of Rome (Italy) using the next-generation sequencing on the Illumina MiSeq platform (San Diego, CA, USA) as recommended by the manufacturer. The library sizes had peaks centered from 900 to 1000 bp.

The reads were de novo assembled into contigs using AByss, version 1.5.2 (k parameter = 63) [22]. Contigs longer than 500 bp were selected using an ad hoc script and kept for further analysis. The final assembly ranged from 44 to 414 (median: 143) contigs per sample (N50: 335,064–64,441; median: 111,289). Contigs were merged through the Minimus2 software (Sommer et al., 2007) and resulting DNA sequences were analysed for similarity using the database sequences by the Standard Nucleotide BLAST program (http://blast.ncbi.nlm.nih.gov/Blast.cgi). Illumina-generated sequence data for the whole data set of this study have been deposited at NCBI (BioProject id: PRJNA515715 and accession number SUB5047324).

### Analysis of virulome and resistome

Virulome of all *K. pneumoniae* sample was analysed using the VirulenceFinder-1.4 tool and the Pasteur *K. pneumoniae* database. The resistome of carbapenem-resistant *K. pneumoniae* was analysed using the ResFinder-2.1 software (default identity thresholds [ID] 98%) which was provided by the Center for Genomic Epidemiology (http://www.genomicepidemiology.org) and the resources of the Pasteur MLST *K. pneumoniae* database.

### Multilocus sequence typing (MLST) in silico

he in silico MLST analysis was made by comparing the whole-genome sequences against the *K. pneumoniae* alleles profiles available at http://www.pasteur.fr/mlst (Genotyping of Pathogens and Public Health, Institute Pasteur, Paris, France).

### Core single-nucleotide polymorphisms (SNPs)

Phylogenetic analysis based on genome-wide single nucleotide polymorphisms (SNPs) were conducted detecting SNPs through the kSNP v2.1.2 program (*k*-mer = 21), which defines a SNP locus as an oligo of length *k* surrounding a central SNP allele [26]. Maximum likelihood tree based on the 57,766 core SNPs identified by kSNP program was visualised using the Dendroscope v3.2.10 software [27]. Strain 8S, *K. pseudopneumoniae*, was used as outgroup to root the tree.

### Statistical analysis

Data were expressed as absolute numbers or percentages. The Chi-squared test was used to compare proportions (as appropriate). Values of *p* < 0.05 were considered statistically significant. The statistical analysis were performed with MedCalc Statistical software version 16.8 (MedCalc Software bvba, Ostend, Belgium; https://www.medcalc.org; 2016).

## Results

**Fig. 1:**
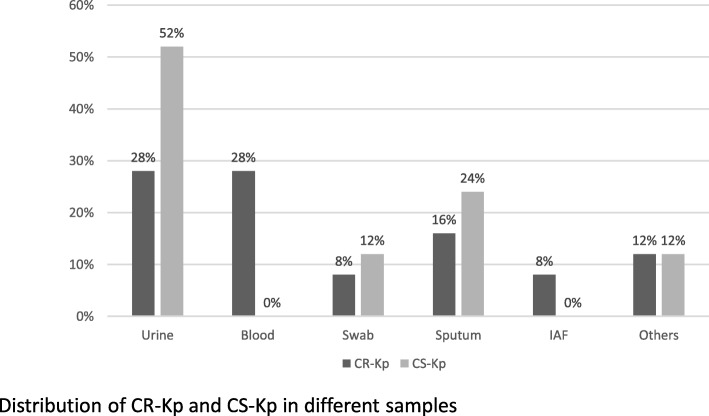
Samples from where isolated the strains

CR-Kp were mainly isolated from blood and urinary samples (28% for both) (Fig. 1), while the ward from which CR-Kp was primarily isolated was the Anaesthesia and Resuscitation Department (24%) (Table 1). Regarding CS-Kp, urines were the major isolation sample (52%) (Fig. 1), while the ward from which CS-Kp was primarily isolated was the Respiratory Department (52%) (Table 1).

### Antibiotic resistance

The percentages of antibiotic resistance among *K. pneumoniae* carbapenem-resistant and carbapenem-susceptible strains are reported in Table 2. As shown, the CR-Kp group displayed a higher percentage of resistance for all tested antibiotics compared to the susceptible group. Statistical significance was calculated where applicable. In particular, 20% of CR-Kp and 4% of CS-Kp were colistin-resistant. Complete antibiotic resistance profile of CR-Kp are shown in Additional file 1.

**Table 2:** Percentage of antibiotic resistance in carbapenem resistant and susceptible *K. pneumoniae*

Class	Antibiotics	*K. pneumoniae* CR %	*K. pneumoniae* CS %	*P* value
Aminoglicosydes	Gentamycin	64	48	0.022
Carbapenems	Imipenem	100	0	NA
	Meropenem	100	0	NA
	Ertapenem	100	0	NA
Monobactams	Aztreonam	100	60	NA
Fluoroquinolones	Ciprofloxacin	100	64	NA
Sulfonamides- Trimethoprim	Trimethoprim-sulfamethoxazole	76	60	0.015
Penicillin	Amoxicillin/ clavulanic acid	100	56	NA
	Piperacillin/tazobactam	100	44	NA
Cephalosporin	Cefotaxime	100	60	NA
	Cefuroxime	100	60	NA
	Cefepime	88	56	0
	Ceftazidime	100	60	NA
	Fosfomycin c/G6P	36	16	0.001
Tetracyclin	Tigecyclin	8	4	0.233
Colistin		20	4	0

NA: chi-squared test not applicable

### MLST analysis and Core single-nucleotide polymorphisms (SNPs) phylogenetic analysis

**Fig. 2:**
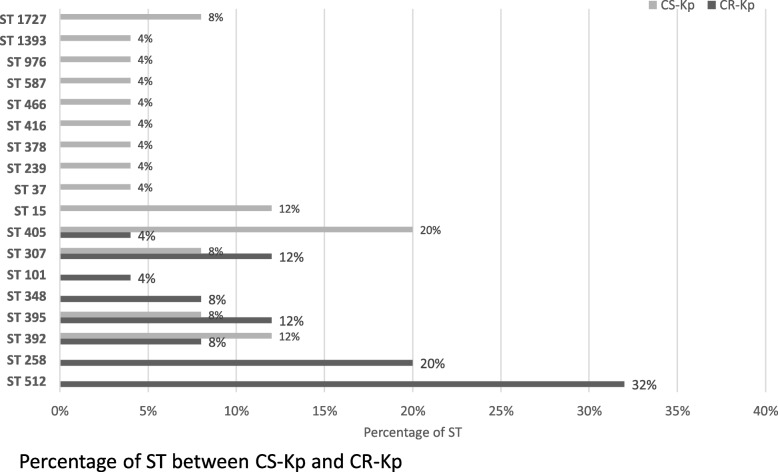
Percentage of ST found

MLST in silico analysis of CR-Kp revealed that 52% belonged to CC258. In particular, 5 strains were ST258 (20%) and 8 were ST512 (32%). The remaining 12 strains were distributed as follows: 3 were ST395 (12%), 3 were ST307 (12%), 2 were ST392 (8%), 2 were ST348 (8%), 1 was ST405 (4%) and 1 was ST101 (4%). Among CS-Kp’s we detected 14 different STs. ST405 was the most represented (5 strains, 20%), followed by ST392 and ST15 (3 strains, 12%), ST395, ST307 and ST1727 (2 strains, 8%), and one strain each for all other STs. Figure 2 shows ST distribution across the entire sample.

The core-SNP phylogenetic analysis showed that strains belonging to the same ST clustered in the same groups, without regard to CR susceptibility or resistance (see Additional file 3). Moreover, the SNP analysis allows distinguishing each different strain.

### Virulome: CR-Kp group versus CS-Kp group

Virulence factors distribution in CR-Kp and CS-Kp is detailed in Table 3. The *mrk* operon, which encodes type 3 fimbriae, was detected in all isolates. The *wzi* gene, involved in the capsule attachment to the host cell surface and used for the prediction of capsular (K) antigen type, was found in all CR-Kp isolates (100%), in particular, 3 strains carried the *wzi-2* allele (see Additional file 4). Regarding the iron acquisition systems, the distribution of genes encoding for the yersiniabactin system (*ybt*), its receptor fyuA and the aerobactin system did not show significant differences between CR-Kp and CS-Kp (Table 3). *Klebsiella* ferrous iron uptake system (*kfuA, kfuB* and *kfuC* genes), the two-component system *kvg*AS and the microcin E495 were significantly (*p* < 0.05) prevalent in the CS-Kp group (28%) compared to the CR-Kp one (4%) (Table 3).

**Table 3:** Distribution of virulence factors in carbapenem resistant and susceptible *K. pneumoniae* strains

Virulence factors	Locus-Genes	*K. pneumoniae* CR n (%)	*K. pneumoniae* CS n (%)	P value
Type 3 fimbries	*mrk* operon	25 (100%)	25 (100%)	1.000
Capsule	*wzi*	25 (100%)	23 (92%)	0.552
Iron acquisition systems	*ybt* operon	10 (40%)	15 (60%)	0.089
	Aerobactin iron acquisition siderophore (*iucABCD*)	3 (12%)	3 (12%)	1.000
	Klebsiella Ferric ionic-uptake system (*kfuABC*)	1 (4%)	7 (28%)	0.020
Two-component system	kvgAS	1 (4%)	7 (28%)	0.020
Bacteriocin	Microcin E492	1 (4%)	7 (28%)	0.020

Table 4 displays the comparative analysis of virulence determinants between CR-Kp and CS-Kp belonging to the same ST. All strains belonging to ST405, regardless of their resistance profile, had the same determinants (*mrk*, *wzi*-143, *ybt*, microcin E495 and *kvg*AS. Regarding the strains belonging to ST307, both CR-Kp and CS-Kp strains had the *mrk* operon, *wz*i-173 and ybt operon, with the exception of the 5R isolate, lacking *ybt* genes (Table 4).

**Table 4:** Distribution of virulence determinants in STs clone of *K. pneumoniae* CR and CS

STs	CR-Kp	CS-Kp
ST395	*mrk*, *wzi*-2, *ybt*, yuc,	*mrk*, *wzi*-2, *ybt*, *yuc*
ST307	*mrk*, wzi-173 *ybt* ^1^	*mrk*, *wzi*-173, *ybt*
ST392	*mrk*, wzi-187	*mrk*, *wzi*-187
ST405	*mrk*, wzi-143, *ybt*, kvgAS, E495	*mrk*, *wzi*-143, *ybt*, kvgAS, E495

^1^missing in one of the three CR isolates

### Virulome subanalysis by STs across the CR-Kp group

CR-Kp isolates belonging to ST512, ST258 did not carry virulence determinants other than *mrk* and *wzi* (Table 4). The three strains belonging to ST395 carried four virulence determinants: *mrk*, *fyu*A/*irp2*, iucABCD and *wzi* type 2. *wzi* gene type 27 was found in the two ST392 isolates together with the *mrk* operon. Strains belonging to ST348 carried the *mrk* operon, *ybt* operon and *wzi*-94 gene, whereas the ST101 strain exhibited *mrk*, *ybt* operon, *wzi* type 17 and the kfuABC system. The ST405 isolate was the only one that carried the kvgAS operon and the microcin E495, together with the *mrk* operon, *ybt* operon and the *wzi-*143.

### Resistome analysis across the CR-Kp group

The in silico β-lactamase characterisation of CR-Kp isolates showed that the most frequent carbapenemase-producing gene was *bla*SHV (100%). In particular, SHV variant 182 was detected in 16 out of 25 isolates (64%), while variant 28 in four strains (16%), three ST307 and the only ST101 isolate. *bla*KPC was identified in 88% of isolates and the most common variant was *bla*KPC-3 (90.9%). *bla*TEM was found in 23 isolates (92%), all of which were variant 1 and *bla*CTX-M (variant 15) was found in 8 isolates (32%). *bla*OXA was found in 22 isolates (88%), 14 of these were variant 9 (63.6%), 5 were variant 1 (22.72%) and 3 isolates (13.63%) presented both variants, *bla*OXA-1 and *bla*OXA-9. Five isolates, one belonging to ST395, two to ST307, one to ST101 and one to ST405 owned all the five carbapenemase-producing genes investigated (Table 5). Complete data from the in silico analysis (e.g. encoding efflux pumps, heavy metal resistance system, genes involved in aminoglycoside and fluoroquinolone resistance) are shown in Additional file 2.

**Table 5:** *K. pneumoniae* CR profile: colistin susceptibility, carbapenemase, ESBL and beta-lactamases genes

STs	ID	CS (mg/L)	bla KPC	bla SHV	bla CTX-M	bla TEM	bla OXA
ST512	6 R	<=1	3	182	–	1	9
	7 R	<=1	3	182	–	1	9
	8 R	<=1	3	182	–	–	–
	11 R	<=1	3	182	–	1	9
	16 R	<=1	3	182	–	1	9
	19 R	> 4	3	182	–	–	1
	22 R	<=1	3	182	–	1	9
	25 R	<=1	3	182	–	1	9
ST258	12 R	<=1	3	182	–	1	9
	18 R	<=1	3	182	–	1	9
	21 R	<=1	3	182	–	1	9
	23 R	> 4	3	182	–	1	9
	24 R	<=1	3	182	–	1	9
ST395	2 R	<=1	3	182	–	1	–
	4 R	<=1	3	182	15	1	1
	10 R	<=1	3	182	–	1	–
ST307	5 R	<=1	9	28	15	1	9
	13 R	<=1	3	28	15	1	1/9
	20 R	> 4 R	2	28	–	1	1/9
ST392	1 R	<=1	3	67	15	1	9
	3 R	<=1	–	67	15	1	1
ST 348	15 R	> 4 R	–	81	15	1	1
	17 R	<=1	–	81	15	1	1
ST 101	9 R	<=1	3	28	–	1	9
ST 405	14 R	> 4 R	3	76	15	1	1/9

## Discussion

The epidemiology of CR-Kp in our geographic area (Palermo, Italy) has already been characterized in the late 2008 at the emergence of CR-Kp ST258 clones [20]. However, several reports have suggested an ongoing epidemiological change in the last years. In fact, whereas CC258 (ST258 and ST512) is still prevalent, several other STs are emerging and circulating [23, 24, 28].

This study set out with the aim of assessing the current dissemination and genetic characteristics of *K. pneumoniae* in Palermo. Even though a larger sample may have allowed to gain more representative data, our preliminary data reveal a complex situation characterized by: 1) high genome “plasticity” of both CR-Kp and CS-Kp, due to the presence of several virulence and resistance determinants carried by mobile genetic elements; 2) a CR-Kp group showing an important genetic diversity of lineages, with 8 different STs identified; 3) an overlapping of multi-drug resistance and hyper virulence traits in the CR-Kp group.

Regarding STs, our comprehensive analysis indicates that, although ST258 and ST512 remain the most representative ones, other STs (e.g. ST307, ST395, ST392, ST348, ST405 and ST101) have been detected in our area. These results are consistent with the surveillance data from other authors [20–24, 28]. Moreover, our findings on β-lactamase characterisation showed that the *bla*SHV gene was the most commonly found in our sample, followed by *bla*TEM and *bla*KPC, deviating from other studies [11, 29, 30], while respect to the KPC-type enzyme our results are in line with those of other studies - as the most commonly encountered is *bla*KPC-3 [21, 23, 31–33]. Regarding STs, the most represented across the CR-Kp group were ST512 and ST258. These isolates did not carry any distinguishing virulence determinant (except for the *mrk* operon and *wzi* gene, which were present in all the samples), suggesting that the success of these clones may only depend on the acquisition of the *bla*KPC gene [9, 15, 34].

Among the ST512 isolates, we found the 19R strain profile to be particularly interesting. This colistin-resistant strain was the only one in its ST group that showed both the yersiniabactin system and its receptor, which has been detected in several *K. pneumoniae* MDR clones; despite of this, the clinical effect of the yersiniabactin system on CR-Kp infections has not been clearly determined [4, 35, 36].

Our data also match those of reports suggesting the recent spread of the well-known clone ST307. In fact, in 2014, a CR-Kp ST307 clone carrying the *bla*KPC-3, coproducing the *bla*CTX-M-15, has been isolated in three Palermo’s Hospitals [23]. The virulome analysis of our ST307 strains has revealed, that 5R isolate was the only one missing the yersiniabactin locus in its ST group. Furthermore, 13R and 5R isolates, two of the three strains belonging to ST307, showed the co-presence of all five carbapenem resistance genes analysed. The co-presence of the detected virulence factors together with the MDR phenotype may explain the diffusion of this clone and the severity of its infections, which have been reported as characterised by higher mortality rates (over 50%) compared to other clones [37, 38]. Moreover, leaving aside specific considerations about the clone ST307, the co-presence of five carbapenem resistance genes that we detected in five strains of our sample (20%) is in line with that of a study by Ferreira et al., which has recently reported that 72% of the *K. pneumoniae* isolated from a Brazilian Intensive care Unite co-produced *bla*KPC, *bla*OXA, *bla*TEM, *bla*SHV, and *bla*CTX-M [39].

Another emerging clone, already isolated by other authors in Palermo and also detected in our CR-Kp sample, was the ST395 [40]. Strains belonging to this clone presented the *wzi*2 allele, which encodes the type K2 capsular antigen that represents one of the most virulent serotypes, thus defined “more virulent” [41, 42]. CR-Kp ST395 strains also carried the yersiniabactin system and its receptor and were the only resistant isolates to possess the aerobactin system (iucABCD). Two aspects of these strains should be addressed here: i) 4R isolate showed the co-presence of five carbapenem resistance genes; ii) to the best of our knowledge, this is the first time that a CR-Kp ST395 clone is reported as carrying a type 2 capsule. This finding was unexpected and seems to be in contrast with the concept that MDR and hyper virulent clonal complexes do not normally overlap [42].

Moreover, our data confirm the spreading of the ST392 clone. A recently published study by Di Mento and colleagues has reported, for the first time, the isolation of a *K. pneumoniae* strain ST392 *bla*KPC-3 carrying the *bla*CTX-M-15, *bla*SHV-11 and *bla*TEM-1 genes from a patient in Palermo who had undergone kidney-pancreas transplantation [43]. In our CR-Kp ST392 sample, the 1R strain which was isolated from blood at the General and Emergency Surgery Department in 2015, showed the co-existence of *bla*KPC-3, *bla*SHV-67, *bla*CTX-M-15, *bla*TEM-1 and *bla*OXA-9. This result is interesting as the only other KPC-producing *K. pneumoniae* ST392 ever reported was isolated in China but with a different isoform, KPC-2 [44]. However, it is important to underline that the other ST392 strain (3R) in our CR-Kp sample did not carry the *bla*KPC gene, suggesting that the ST392 KPC-3 clone may have acquired the resistance gene through horizontal transmission, as described by other authors [43].

This study also revealed two MDR CR-Kps belonging to ST348 and carrying the *bla*CTX-M-15, *bla*SHV-81, *bla*TEM-1 and *bla*OXA-1 genes. Strains belonged to ST348, but harboured the *bla*KPC-3, which had been previously reported as responsible of several epidemic events in Portugal [45]. Considering that one of our isolates was colistin-resistant and the ease with which *K. pneumoniae* acquires the *bla*KPC gene, we can consider the MDR CR-Kp ST348 strains as possibly emerging high-risk clones.

Three other important data that emerge from our results and complete the description of the CR-Kp epidemiological scene in Palermo are: firstly, the circulation of strains belonging to ST101. This clone was previously recognized worldwide as a high risk carbapenem-producing clone [30] and has already been identified in Palermo and in the North of Italy [46–48]. Our ST101 strain (9R) carried the *bla*KPC-3, *bla*SHV-28, *bla*TEM-1 and *bla*OXA-9 genes and the *Klebsiella* ferrous uptake system, which is typically found in *K. pneumoniae* hypervirulent strains [49, 50]; secondly, the characteristic of the strain belonging to ST405, which carried the *bla*KPC-3, *bla*CTX-15, *bla*SHV-76, *bla*TEM-1 and *bla*OXA-1/9 genes, the *aac6-Ib-cr* and *qnrB* and was resistant to colistin. Strains from ST405 have similarly caused an outbreak in a Spanish Hospital neonatal unit [50] and have already been isolated in Palermo [24]. It is important to underline that the genes involved in microcin production and kvgA/S system were detected in all isolates belonging to ST405, both resistant and susceptible to carbapenem, possibly indicating a stable and characteristic genetic pattern for these clones; lastly to the best of our knowledge this is the first study to report in Italy the isolation of five CR-Kp isolates belonging to different STs showing the co-presence of five carbapenem resistance genes.

## Conclusions

These results are significant in at least two major respects. Overall, this study strengthens the idea that the epidemiological frame in the Palermo area (Sicily, Italy) is shifting and new MDR clones are emerging. However, our analysis, which included the comparison of the virulence degree of CS-Kp and CR-Kp isolates, has unexpectedly revealed that the latter are acquiring highly-virulent determinants and the co-presence of more resistance genes. Undoubtedly, since co-existence of antibiotic resistance and virulence factors may lead to life-threatening untreatable and invasive *K. pneumoniae* infections, this is an important issue to take into consideration for future genomic surveillance studies.

## Supplementary information

**Additional file 1. ***K. pneumoniae* CR antibiotic resistance profile. Results of antibiotic resistance assay of *K. pneumoniae* CR.

**Additional file 2. ***K. pneumoniae* CR in silico analysis of resistome and virulome. Results of in silico analysis of sequences encoding for efflux pumps, heavy metal resistance system, and genes involved to aminoglycoside and fluoroquinolone resistance.

**Additional file 3. ***wzi* analysis of CR-K and CS-K. Table of contig and allele of wzi gene in *K. pneumoniae* CR and CS.

**Additional file 4.** SNP phylogenetic tree. Core Single-Nucleotide Polymorphisms dendrogram.

## Data Availability

The dataset used and analyzed during the current study are available from the corresponding author on reasonable request.

## Abbreviations

CR-Kp: Carbapenem-resistant *K. pneumoniae*
CS-Kp: Carbapenem-susceptible *K. pneumoniae*
NGS: Next generation sequencing
MLST: Multi-locus sequence typing
SNP: Core single nucleotide polymorphism
CC: Clonal complex
ST: Sequence type
WHO: World Health Organization
CDC: US Centers for Disease Control and Prevention
MDR: Multi drug resistant
