# Supplementary material for: Co-existence of virulence factors and antibiotic resistance in new Klebsiella pneumoniae clones emerging in south of Italy
Source: BMC Infect Dis. 2019 Nov 4;19:928. doi: 10.1186/s12879-019-4565-3 (PMC6829812; doi:10.1186/s12879-019-4565-3)
Supplement: Supplementary file 1 — Additional file 1. K. pneumoniae CR antibiotic resistance profile. Results of antibiotic resistance assay of K. pneumoniae CR. [file 12879_2019_4565_MOESM1_ESM.docx]

| S1 *K. pneumoniae* CR antibiotic-resistance profile | | | | | | | | | | | | | | | | |
| --- | --- | --- | --- | --- | --- | --- | --- | --- | --- | --- | --- | --- | --- | --- | --- | --- |
| Isolate | **CN** | **IMI** | **MEP** | **ETP** | **CIP** | **SXT** | **TGC** | **CS** | **CTX** | **CXM** | **AUG** | **TZP** | **FEP** | **CAZ** | **ATM** | **FOS** |
| 1 R | <=1 | >8 | >1 | >8 | >1 | >4/76 | 1 | <=1 | >4 | >8 | >8/2 | >16/4 | >8 | >8 | >16 | 64 |
| 2 R | 2 | >8 | >1 | >8 | >1 | 4/76 | 1 | <=1 | >4 | >8 | >8/2 | >16/4 | <=1 | >8 | >16 | 32 |
| 3 R | >4 | 8 | >1 | 4 | >1 | >4/76 | 0.5 | <=1 | >4 | >8 | >8/2 | >16/4 | >8 | >8 | >16 | <=16 |
| 4 R | >4 | >8 | >8 | >1 | >1 | 2/38 | 1 | <=1 | >4 | >8 | >32/2 | >16/4 | >8 | >8 | >16 | 32 |
| 5 R | >4 | >8 | >1 | >8 | >1 | >4/76 | 0.5 | <=1 | >4 | >8 | >8/2 | >16/4 | >8 | >8 | >16 | 64 |
| 6 R | 4 | >8 | >1 | >8 | >1 | <=1/19 | 0.5 | <=1 | >4 | >8 | >8/2 | >16/4 | <=1 | >8 | >16 | <=16 |
| 7 R | >4 | >8 | >1 | >8 | >1 | >4/76 | 2 | <=1 | >4 | >8 | >8/2 | >16/4 | >8 | >8 | >16 | <=16 |
| 8 R | 2 | >8 | >1 | >8 | >1 | <=1/19 | 0.5 | <=1 | >4 | >8 | >8/2 | >16/4 | <=1 | >8 | >16 | >64 |
| 9 R | <=1 | >8 | >1 | >8 | >1 | <=1/19 | 0.5 | <=1 | >4 | >8 | >8/2 | >16/4 | >8 | >8 | >16 | <=16 |
| 10R | 4 | >8 | >1 | >8 | >1 | 2/38 | 1 | <=1 | >4 | >8 | >8/2 | >16/4 | >8 | >8 | >16 | 64 |
| 11 R | >4 | >8 | >1 | >8 | >1 | >4/76 | 0.5 | <=1 | >4 | >8 | >8/2 | >16/4 | >8 | >8 | >16 | <=16 |
| 12 R | 4 | >8 | >1 | >8 | >1 | >4/76 | 1 | <=1 | >4 | >8 | >8/2 | >16/4 | >8 | >8 | >16 | >64 |
| 13 R | >4 | 8 | >8 | >1 | >1 | >4/76 | 1 | <=1 | >4 | >8 | >8/2 | >16/4 | >8 | >8 | >16 | <=16 |
| 14 R | >4 | >8 | >8 | >1 | >1 | >4/76 | >2 | >4 | >4 | >8 | >8/2 | >16/4 | >8 | >8 | >16 | 32 |
| 15 R | >4 | >8 | >8 | >1 | >1 | >4/76 | 1 | >4 | >4 | >8 | >8/2 | >16/4 | >8 | >8 | >16 | 32 |
| 16 R | 2 | >8 | >8 | >1 | >1 | >4/76 | 1 | <=1 | >4 | >8 | >32/2 | >16/4 | >8 | >8 | >16 | <=16 |
| 17 R | 2 | >8 | >8 | >1 | >1 | >4/76 | 1 | <=1 | >4 | >8 | >32/2 | >16/4 | >8 | >8 | >16 | >64 |
| 18 R | 2 | 8 | >8 | >1 | >1 | >4/76 | <=0.5 | <=1 | >4 | >8 | >32/2 | >16/4 | >8 | >8 | >16 | >64 |
| 19R | >4 | >8 | >8 | >1 | >1 | >4/76 | 1 | >4 | >4 | >8 | >32/2 | >16/4 | >8 | >8 | >16 | 32 |
| 20 R | <=1 | >8 | >8 | >1 | >1 | >4/76 | <=0.5 | >4 | >4 | >8 | >32/2 | >16/4 | 8 | >8 | >16 | 32 |
| 21 R | 2 | 8 | >8 | >1 | >1 | >4/76 | <=0.5 | <=1 | >4 | >8 | >32/2 | >16/4 | >8 | >8 | >16 | <=16 |
| 22 R | 4 | >8 | >8 | >1 | >1 | <=1/19 | 1 | <=1 | >4 | >8 | >32/2 | >16/4 | >8 | >8 | >16 | >64 |
| 23 R | >4 | >8 | >8 | >1 | >1 | >4/76 | <=0.5 | >4 | >4 | >8 | >32/2 | >16/4 | >8 | >8 | >16 | 32 |
| 24R | 4 | >8 | >8 | >1 | >1 | >4/76 | <=0.5 | <=1 | >4 | >8 | >32/2 | >16/4 | >8 | >8 | >16 | >64 |
| 25 R | 4 | >8 | >8 | >1 | >1 | >4/76 | <=0.5 | <=1 | >4 | >8 | >32/2 | >16/4 | >8 | >8 | >16 | <=16 |
| CN: Gentamycin; IMI: Imipenem; MEP: Meropenem; ETP: Ertapenem; CIP: Ciprofloxacin; SXT: Trimethoprim-sulfamethoxazole; TGC: Tigecyclin; CS: colistin; CTX: Cefotaxime; CXM: Cefuroxime; AUG: Amoxicillin/ clavulanic acid; TZP: Piperacillin/tazobactam; FEP: Cefepime; CAZ: Ceftazidime; ATM: Aztreonam; FOS: Fosfomycin c/G6P | | | | | | | | | | | | | | | | |
