# Supplementary material for: Co-existence of virulence factors and antibiotic resistance in new Klebsiella pneumoniae clones emerging in south of Italy
Source: BMC Infect Dis. 2019 Nov 4;19:928. doi: 10.1186/s12879-019-4565-3 (PMC6829812; doi:10.1186/s12879-019-4565-3)
Supplement: Supplementary file 2 — Additional file 2. K. pneumoniae CR in silico analysis of resistome and virulome. Results of in silico analysis of sequences encoding for efflux pumps, heavy metal resistance system, and genes involved to aminoglycoside and fluoroquinolone resistance. [file 12879_2019_4565_MOESM2_ESM.docx]

| **S2** *K. pneumoniae* CR *in silico* analysis of resistome and virulome | | | | | | | | | | | | | | | | | | | |
| --- | --- | --- | --- | --- | --- | --- | --- | --- | --- | --- | --- | --- | --- | --- | --- | --- | --- | --- | --- |
|  | | **Efflux resistance systems** | | | | | | | | | | **Heavy metal resistance** | | **Quinolone resistance** | | | **Aminoglycoside resistance** | | |
| **ST** | **ID** | ***acrABR*** | ***envR*** | ***fis*** | ***marA/R*** | ***oqx*** | ***ram*** | ***rarA*** | ***rob*** | ***sdiA*** | ***soxS*** | ***pco/sil*** | ***terE*** | ***gyrA/B*** | ***parC/E*** | ***qnrB/S*** | ***AACs*** | ***ANTs*** | ***APHs*** |
| **ST512** | 6 R | acrA/B/R |  |  |  | oqxA/B/R | ramA/R |  |  |  |  |  |  | gyrA/B | parC/E |  | Aac6pIb |  |  |
|  | 7 R | acrA/B/R |  |  |  | oqxA/B/R | ramA/R |  |  |  |  |  |  | gyrA/B | parC/E |  | Aac6pIb | Ant3ppIa | Aph3pIa |
|  | 8 R | acrA/B/R |  |  |  | oqxA/B/R | ramA/R |  |  |  |  |  |  | gyrA/B | parC/E |  | Aac6pIb | Ant3ppIa |  |
|  | 11 R | acrA/B/R |  |  |  | oqxA/B/R | ramA/R |  |  |  |  |  |  | gyrA/B | parC/E |  | Aac6pIb |  | Aph3pIa |
|  | 16 R | acrA/B/R |  |  |  | oqxA/B/R | ramA/R |  |  |  |  |  |  | gyrA/B | parC/E |  | Aac6pIb |  | Aph3pIa |
|  | 19 R | acrA/B/R |  |  |  | oqxA/B/R | ramA/R |  |  |  |  |  |  | gyrA/B | parC/E |  | Aac3 IIa |  | Aph3pIa |
|  | 22 R | acrA/B/R |  |  |  | oqxA/B/R | ramA/R |  |  |  |  |  |  | gyrA/B | parC/E |  | Aac6pIb | Ant3ppIa |  |
|  | 25 R | acrA/B/R |  |  |  | oqxA/B/R | ramA/R |  |  |  |  |  |  | gyrA/B | parC/E |  | Aac6pIb | Ant3ppIa | Aph3pIa |
| **ST258** | 12 R | acrA/B/R |  |  |  | oqxA/B/R | ramA/R |  |  |  |  |  |  | gyrA/B | parC/E |  | Aac6pIb | Ant3ppIa | Aph3pIa |
|  | 18 R | acrA/B/R |  |  |  | oqxA/B/R | ramA/R |  |  |  |  |  |  | gyrA/B | parC/E |  |  |  | Aph3pIa |
|  | 21 R | acrA/B/R |  |  |  | oqxA/B/R | ramA/R |  |  |  |  |  |  | gyrA/B | parC/E |  | Aac6pIb |  | Aph3pIa |
|  | 23 R | acrA/B/R |  |  |  | oqxA/B/R | ramA/R |  |  |  |  |  |  | gyrA/B | parC/E |  | Aac6pIb |  | Aph3pIa |
|  | 24 R | acrA/B/R |  |  |  | oqxA/B/R | ramA/R |  |  |  |  |  |  | gyrA/B | parC/E |  | Aac6pIb |  |  |
| **ST 395** | 2 R | acrA/B |  |  |  | oqxA/B/R |  |  |  |  |  |  |  | gyrA/B | parC/E | qnrS | Aac6pIb |  |  |
|  | 4 R | acrA/B/R |  |  |  | oqxA/B/R | ramA/R |  |  |  |  |  |  | gyrA/B | parC/E | qnrS | Aac6pIb | Ant2ppIa  Ant3ppIa |  |
|  | 10 R | acrA/B |  |  |  | oqxA/B/R |  |  |  |  |  |  |  | gyrA/B | parC/E | qnrS | Aac6pIb |  |  |
| **ST 307** | 5 R | acrB/R |  |  |  | oqxR | ramA |  |  |  |  |  |  | gyrA/B | parC/E | qnrB/S | Aac3Iva | Ant3ppIa | Aph3ppIb  Aph4Id  Aph6Id |
|  | 13 R | acrB/R |  |  |  | oqxR | ramA/R |  |  |  |  |  |  | gyrA/B | parC/E | qnrB | Aac6pIb  Aac3 IIa |  | Aph3ppIb  Aph6Id |
|  | 20 R | acrB/R |  |  |  | oqxR |  |  |  |  |  |  |  | gyrA/B | parC/E | qnrB | Aac6pIb |  | Aph3ppIb  Aph6Id |
| **ST 392** | 1 R | acrR |  |  |  | oqxA/R | ramA/R |  |  |  |  |  |  | gyrA/B | parC/E | qnrB |  |  | Aph3ppIb  Aph6Id |
|  | 3 R | acrR |  |  |  | oqxA/R | ramA/R |  |  |  |  |  |  | gyrA/B | parC/E | qnrB | Aac6pIb  Aac6pIIa |  | Aph3ppIb  Aph6Id |
| **ST 348** | 15 R | acrA/R |  |  |  | oqxR | ramA/R |  |  |  |  |  |  | gyrA/B | parC | qnrB | Aac6pIb |  | Aph3ppIb  Aph6Id |
|  | 17 R | acrA/R |  |  |  | oqxR | ramA/R |  |  |  |  |  |  | gyrA/B | parC | qnrB | Aac6pIb |  | Aph3ppIb  Aph6Id |
| **ST 101** | 9 R | acrA/B/R |  |  |  | oqxA/B/R | ramA |  |  |  |  |  |  | gyrB | parC |  |  |  |  |
| **ST 405** | 14 R | acrR |  |  |  | oqxR |  |  |  |  |  |  |  | gyrA/B | parC |  | Aac6pIb  Aac3 IIa |  | Aph3ppIb  Aph6Id |
