# Supplementary figures and images for: Co-existence of virulence factors and antibiotic resistance in new Klebsiella pneumoniae clones emerging in south of Italy
Source: BMC Infect Dis. 2019 Nov 4;19:928. doi: 10.1186/s12879-019-4565-3 (PMC6829812; doi:10.1186/s12879-019-4565-3)

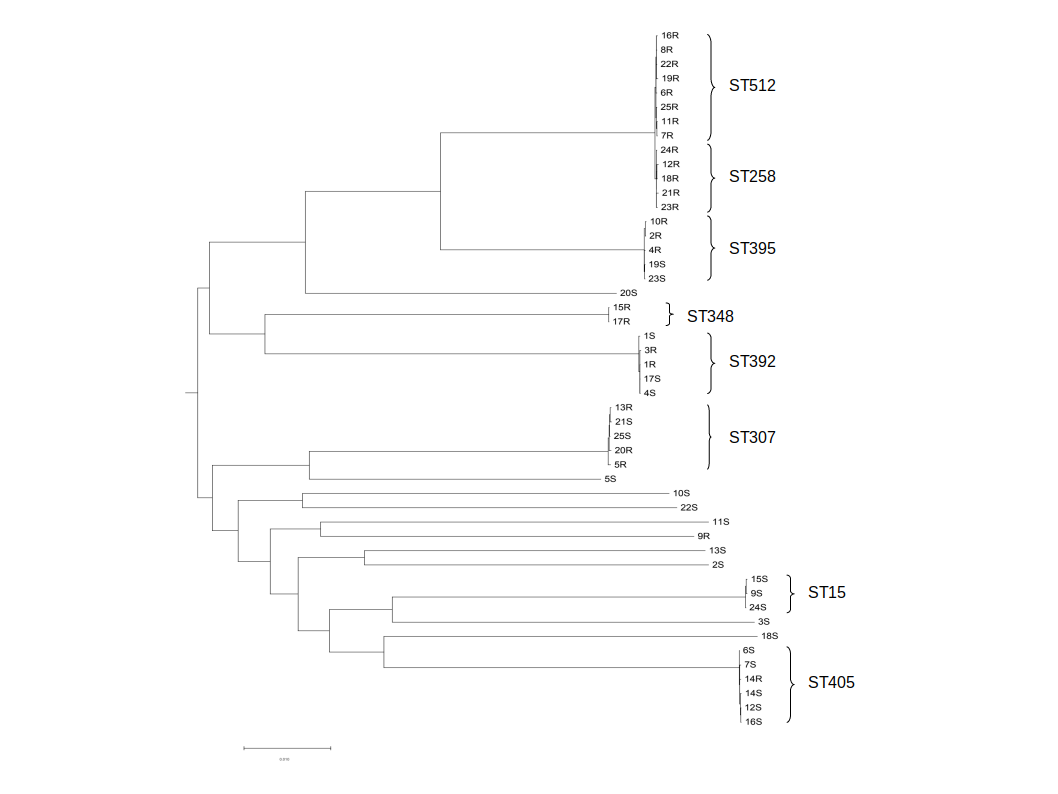


**S3.** SNP phylogenetic tree

0.050

Supplement: Supplementary file 3 — Additional file 3. wzi analysis of CR-K and CS-K. Table of contig and allele of wzi gene in K. pneumoniae CR and CS. [file 12879_2019_4565_MOESM3_ESM.docx]
