# Supplementary material for: Co-existence of virulence factors and antibiotic resistance in new Klebsiella pneumoniae clones emerging in south of Italy
Source: BMC Infect Dis. 2019 Nov 4;19:928. doi: 10.1186/s12879-019-4565-3 (PMC6829812; doi:10.1186/s12879-019-4565-3)
Supplement: Supplementary file 4 — Additional file 4. SNP phylogenetic tree. Core Single-Nucleotide Polymorphisms dendrogram. [file 12879_2019_4565_MOESM4_ESM.docx]

| S4 wzi analysis of CR-K and CS-K | | | | | | |
| --- | --- | --- | --- | --- | --- | --- |
| Strain | **Allele** | **Lenght** | **Contig** | **Start**  **position** | **End position** | **Attributes** |
| 1R | 187 | 447 | NODE_11_length_154730_cov_20.5707_ID_6389 | 146133 | 146579 |  |
| 2R | 2 | 447 | NODE_13_length_105604_cov_22.4893_ID_6521 | 100265 | 100711 | K-type: K2 |
| 3R | 187 | 447 | NODE_126_length_4512_cov_19.4386_ID_6215 | 3210 | 3656 |  |
| 4R | 2 | 447 | NODE_43_length_46408_cov_30.9348_ID_6173 | 40719 | 41165 | K-type: K2 |
| 5R | 173 | 447 | NODE_1_length_295915_cov_36.2954_ID_12068 | 3203 | 3649 |  |
| 6R | 154 | 447 | NODE_34_length_48738_cov_21.6058_ID_5906 | 40817 | 41263 |  |
| 7R | 154 | 447 | NODE_1_length_283253_cov_27.1024_ID_7152 | 277941 | 278387 |  |
| 8R | 154 | 447 | NODE_8_length_213723_cov_29.0834_ID_4817 | 205856 | 206302 |  |
| 9R | 137 | 447 | NODE_5_length_211154_cov_16.6651_ID_4595 | 205833 | 206279 | K-type: K17 |
| 10R | 2 | 447 | NODE_11_length_105555_cov_13.7357_ID_7183 | 100265 | 100711 | K-type: K2 |
| 11R | 154 | 447 | NODE_2_length_285777_cov_34.9216_ID_7730 | 277938 | 278384 |  |
| 12R | 154 | 447 | NODE_11_length_155559_cov_27.3964_ID_7615 | 4865 | 5311 |  |
| 13R | 173 | 447 | NODE_35_length_47685_cov_10.4828_ID_7016 | 6546 | 6992 |  |
| 14R | 143 | 447 | NODE_114_length_3969_cov_21.7264_ID_7349 | 860 | 1306 |  |
| 15R | 94 | 447 | NODE_77_length_18386_cov_27.9362_ID_5758 | 3285 | 3731 |  |
| 16R | 154 | 447 | NODE_124_length_6200_cov_15.3622_ID_10363 | 898 | 1344 |  |
| 17R | 94 | 447 | NODE_95_length_4585_cov_15.7023_ID_5233 | 3246 | 3692 |  |
| 18R | 154 | 447 | NODE_1_length_213626_cov_22.1519_ID_8546 | 205805 | 206251 |  |
| 19R | 154 | 447 | NODE_72_length_19948_cov_27.5186_ID_10218 | 4898 | 5344 |  |
| 20R | 173 | 447 | NODE_4_length_212096_cov_10.6091_ID_8242 | 5849 | 6295 |  |
| 21R | 154 | 447 | NODE_40_length_46350_cov_26.3893_ID_9269 | 5088 | 5534 |  |
| 22R | 154 | 447 | NODE_38_length_46129_cov_26.704_ID_7171 | 40817 | 41263 |  |
| 23R | 154 | 447 | NODE_10_length_115442_cov_18.1885_ID_9001 | 4863 | 5309 |  |
| 24R | 154 | 447 | NODE_8_length_157981_cov_26.7982_ID_6667 | 7428 | 7874 |  |
| 25R | 154 | 447 | NODE_36_length_48755_cov_15.8665_ID_7204 | 7478 | 7924 |  |
| 1S | 187 | 447 | NODE_6_length_264318_cov_33.0314_ID_5333 | 256241 | 256687 |  |
| 2S | 37 | 447 | NODE_11_length_154878_cov_16.8304_ID_4410 | 151269 | 151715 | K-type: K22.37 |
| 3S | 150 | 447 | NODE_44_length_51858_cov_39.3354_ID_4989 | 5844 | 6290 |  |
| 4S | 187 | 447 | NODE_5_length_272271_cov_24.1804_ID_3879 | 263595 | 264041 |  |
| 5S | 27 | 447 | NODE_26_length_49626_cov_37.3489_ID_2531 | 8380 | 8826 | K-type: K27 |
| 6S | 143 | 447 | NODE_3_length_289523_cov_25.1564_ID_4250 | 283274 | 283720 |  |
| 7S | 143 | 447 | NODE_82_length_6535_cov_26.7264_ID_4788 | 904 | 1350 |  |
| 8S | ND |  |  |  |  |  |
| 9 S | 93 | 447 | NODE_48_length_40916_cov_22.047_ID_5335 | 33879 | 34325 | K-type: K60 |
| 10S | ND |  |  |  |  |  |
| 11S | 177 | 447 | NODE_35_length_48510_cov_30.3284_ID_5554 | 40796 | 41242 |  |
| 12S | 143 | 447 | NODE_6_length_219074_cov_30.3613_ID_4397 | 211324 | 211770 |  |
| 13S | 8 | 447 | NODE_2_length_465925_cov_30.7743_ID_2672 | 5335 | 5781 | K-type: K8 |
| 14S | 143 | 447 | NODE_4_length_343372_cov_14.112_ID_4680 | 334262 | 334708 |  |
| 15S | 93 | 447 | NODE_37_length_42132_cov_22.3789_ID_4399 | 33879 | 34325 | K-type: K60 |
| 16S | 143 | 447 | NODE_6_length_289496_cov_31.4279_ID_4371 | 283275 | 283721 |  |
| 17S | 187 | 447 | NODE_7_length_266884_cov_33.2039_ID_3494 | 258119 | 258565 |  |
| 18S | 415 | 447 | NODE_5_length_278208_cov_23.5942_ID_3177 | 271028 | 271474 |  |
| 19S | 2 | 447 | NODE_131_length_5750_cov_45.985_ID_10111 | 4446 | 4892 | K-type: K2 |
| 20S | 96 | 447 | NODE_21_length_81207_cov_34.9348_ID_3900 | 74267 | 74713 | K-type: K38 |
| 21S | 173 | 447 | NODE_2_length_311500_cov_26.9429_ID_5008 | 303486 | 303932 |  |
| 22S | 227 | 447 | NODE_24_length_80102_cov_31.0562_ID_4741 | 4856 | 5302 | K-type: K60 |
| 23S | 2 | 447 | NODE_13_length_105565_cov_37.2736_ID_6182 | 100265 | 100711 | K-type: K2 |
| 24S | 93 | 447 | NODE_49_length_39368_cov_35.4523_ID_4843 | 5048 | 5494 | K-type: K60 |
|  | 173 | 447 | NODE_6_length_209474_cov_23.1484_ID_6052 | 205817 | 206263 |  |
| 25S | 173 | 447 | NODE_6_length_209474_cov_23.1484_ID_6052 | 205817 | 206263 |  |
